# The history of pain measurement in humans and animals

**DOI:** 10.3389/fpain.2022.1031058

**Published:** 2022-09-15

**Authors:** Jeffrey S. Mogil

**Affiliations:** Department of Psychology and Anesthesia, McGill University, Montreal, QC, Canada

**Keywords:** algesiometry, history, human, animal, acute, chronic

## Abstract

Pain needs to be measured in order to be studied and managed. Pain measurement strategies in both humans and non-human animals have varied widely over the years and continue to evolve. This review describes the historical development of human and animal algesiometry.

## Introduction

A common adage, variously attributed to management consultants Peter Drucker or W. Edwards Deming, asserts that “if you can't measure it, you can't manage it.” This is obviously true for pain, which can neither be managed nor even studied without being measured. The measurement of pain in humans and animals—algesiometry—has been a continuing focus of pain researchers since the late 1800s, coinciding with the development of psychophysics (1). Methods for estimating the intensity of a stimulus, applied to human skin, required to evoke the perception of pain were developed using noxious electrical (2), mechanical (3), and heat (4, 5) stimuli. In the first few decades of the 20th century, such methods were used to establish analgesic dose-response curves of opioids and aspirin (6–8), investigate intra- and inter-individual variability (5), circadian rhythmicity (7), spatial summation (9), counter-irritation (5), and even to demonstrate the effect of modulatory factors such as distraction (5). The first algesiometric techniques for laboratory animals were based on these human methods, with noxious stimuli aimed at easily accessible body parts such as the tail (10, 11) and plantar hind paw (12, 13). The von Frey filament test of mechanical sensitivity is used identically in human and non-human animals, with the exception of the range of filament force employed and the nature (verbal or non-verbal) of the response. Of course, refinements of these procedures have been made over the years and continue to this day (e.g., 14–16).

**Figure 1:**
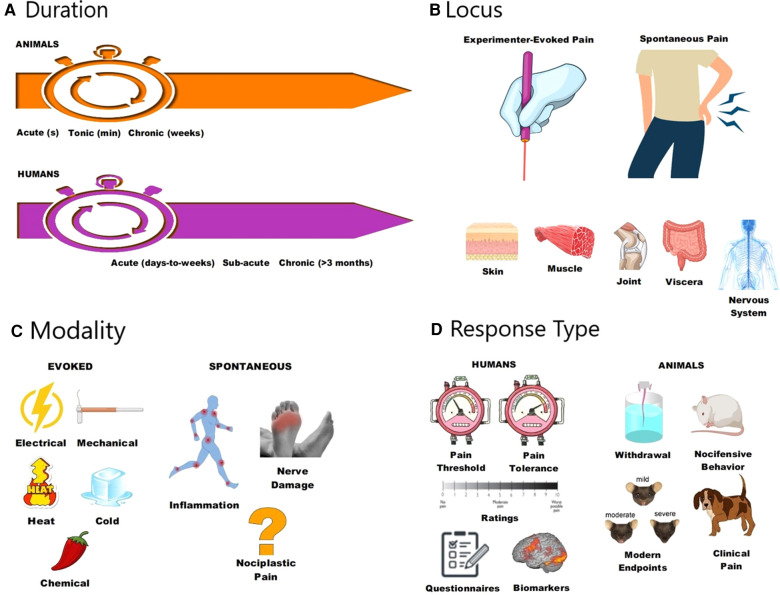
Different ways to think about different types of pain, all with implications for algesiometry in humans and non-human animals. Pain can be dissociated based on its duration (**A**), locus (**B**), modality (**C**), and response type (**D**).

One might ask why any further developments in algesiometry were (and are) required. I would argue that these historical methods, though useful, fail to suffice for a number of reasons best thought of in terms of the dimensions, continua, and categories shown in Figure 1. Likely the most important of these is *duration* (see Figure 1A). All algesiometric methods introduced prior to the mid-1900s are measurements of acute pain, in which the time elapsing from stimulus onset to pain threshold can be measured in seconds, and also in seconds from pain threshold to pain tolerance (or, in animals, to withdrawal from the stimulus). The stimuli need to be of sufficient intensity to potentially cause tissue damage, and Woodworth / Sherrington (17) suggested that “pain” was mediated by higher-order systems driven by these stimuli. Although such stimuli clearly produce an aversive condition that is valid as a painful state (e.g., picking up a hot coffee cup), the pain states that researchers and clinicians are most interested in studying and managing, respectively, occur on a much longer time scale: hours to years. Whether mechanisms and treatment strategies applicable to pain measured in seconds are also applicable to pain measured in months is hardly a given. A second dimension of pain highly relevant to algesiometry is its *locus* (see Figure 1B). The historical algesiometric techniques described above involve noxious stimuli being applied by an experimenter to the skin of the pain-perceiving subject. Again, it's real pain, but different in character from clinical pain, which is usually not superficial but deep (e.g., muscle pain, joint pain, visceral pain) (18), if it can be precisely localized at all, and not evoked by a stimulus external to the body but rather arising within the body itself. The type of pain we really want to understand is *spontaneous* pathological pain (19, but see 20), which may or may not share underlying mechanisms with evoked pain. As already well appreciated in the 1800s, different noxious stimulus *modalities* (see Figure 1C) exist. Pain can be caused by mechanical force, electric shock, heat, and cold. But it can also be evoked by irritant chemicals applied to the skin or otherwise introduced into the body. Much clinical pain arises *via* the presence of inflammation or nerve damage, which are both associated with the release of chemical messengers in the immune and nervous systems. The generation of nociplastic (previously known as functional or idiopathic) pain remains a mystery. The transduction of different pain modalities by nociceptors is dissociable, mediated by different proteins, and there exists considerable evidence that the processing of different modalities of pain is dissociable at more rostral levels of the neuraxis as well. Especially in the context of pain questionnaires, pain associated with different clinical entities may need to be measured in different ways; this is pain's version of the lumping/splitting problem. Finally, the *type of response* (see Figure 1D) can have serious consequences in algesiometry in both humans and animals. In humans, one can choose to measure pain threshold, pain tolerance, or to elicit quantitative ratings of suprathreshold stimuli. Alternatively, one might eschew quantitative measurement and elicit qualitative descriptors of pain *via* questionnaires, or try to avoid self report entirely in favor of an “objective” biomarker. Animals, of course, cannot be instructed to respond at threshold, cannot easily be motivated to hold off responding until tolerance, and are incapable of verbal report. For them, nocifensive and other behaviors are necessary for experimenters to interpret the presence of pain. This is often held as a disadvantage of preclinical pain research, although a human subject rating their back pain “an 8” is equally a behavior requiring interpretation.

The history of algesiometry is the story of attempts by pain researchers to broaden available tools so that they cover more of these continua, into the range that more typifies the clinical problem at hand. This review will attempt to briefly summarize developments over the years in humans and non-human animals, with respect to the dimensions described above.

## Pain measurement in humans

The measurement of experimental pain in humans using controlled noxious stimuli delivered to the skin (superficially or subdermally), muscles, joints, or viscera has continued unabated since the pioneering psychophysical work in the 1800s. In general, regardless of the noxious stimulus employed, attempts are made to quantify either pain threshold, pain tolerance, ratings of pain on structured scales, magnitude estimation (e.g., by cross-modality matching), or measurement of performance (21). Beyond the many issues surrounding confounds and inter- and intra-individual differences, the obvious limitation is that these are ways to measure sensitivity to the particular noxious stimuli delivered, with no obvious relation (except perhaps in certain pain syndromes such as fibromyalgia) to the clinical pain of a patient, although attempts were often made to compare the magnitude of the experimental pain to a patient's clinical pain.

The first major proponent of abandoning the “dolorimeter” approach to measuring clinical pain was Beecher (22), who advanced the notion of measuring clinical pain by its relief, *via* subjective ratings. But how exactly to provide such ratings? Solutions to this problem include the use of numerical rating scales (NRS) with descriptive “pegs” at the ends (e.g., from 0, no pain, to 10, the worst pain imaginable), verbal rating scales (VRS) of category judgments (e.g., mild, distressing, excruciating), and visual analog scales (VAS) (23), in which pain is indicated by marking a spot along a 10-cm continuum. These have been modified for use in pediatric and elderly populations (24, 25), and categories and pegs translated into different languages. As it became increasingly clear that pain was multidimensional—consisting of sensory/discriminative, motivational/affective, and cognitive components—research subjects and patients were increasingly asked for multiple ratings (e.g., 26).

A rather different approach to the problem was developed by Melzack and Torgerson (27, 28) with the McGill Pain Questionnaire, in which pain is rated qualitatively *via* sensory, evaluative, and affective descriptors (e.g., burning, shooting, troublesome, agonizing). Questionnaires are currently used not only to measure different aspects of pain itself, but also to: (1) more thoroughly characterize disease states featuring pain, and their impact on functioning and quality of life (29, 30); (2) help diagnose the presence of particular types of pain, such as neuropathic pain (31); and (3) quantify putative risk factors for chronic pain, such as catastrophizing (32).

Finally, a whole host of observational methods have been developed in which pain is quantified by others. Such methods, valuable especially for use in non-verbal populations, include the FLACC scale for young children (33), in which facial expression, leg position, activity, crying, and consolability are rated on a 0–2 scoring scale, and the Neonatal Facial Coding System (34), in which pain expression is quantified *via* judgments of facial musculature (e.g., brow bulging, eye squeezing). Other observational methods attempt to bypass patient self-report *via* the measurement, for example, of visible behaviors (e.g., guarding, limping, rubbing, sighing) by clinicians (35) or changes in a child's daily behaviors (e.g., playing less, complaining more) by parents (36). Such techniques are obviously susceptible to bias, although individuals are likely biased self-observers as well.

Recently, the use of modern versions of venerable acute pain measures has been enjoying a renaissance, more for the purpose of patient stratification (e.g., 37) than pain quantification *per se*. Known as quantitative sensory testing (QST), the most comprehensive effort has been by a German consortium involving the QST profiling—using 13 different measures of gain and loss of sensory function—of over a thousand patients and non-patients (38, 39). Although different frequencies of sensory abnormalities were observed in different pain disorders (38), it has more recently been shown that QST batteries are far better at quantifying neuropathy itself than neuropathic pain (40, 41).

Ultimately, although the validity and usefulness of self-reported pain, especially by VAS, has been amply demonstrated (42), researchers and clinicians have always longed for an objective measure, or biomarker, that could be used for diagnostic, prognostic, and/or drug development purposes. Although we are counseled to always “trust the patient”, there are obvious (if not necessarily frequent) incentives for both exaggerated and minimized self-reporting. Putative biomarkers over the years have included physiological measurements, blood protein levels, genetic variants, and nervous system electrical (e.g., microneurography, electroencephalography) and metabolic activity [e.g., positron emission tomograph, functional magnetic resonance imaging (fMRI)]. The leading contender as a pain biomarker is likely fMRI, although pain imagers have cautioned against its over-credulous use (43). It seems likely that useful pain biomarkers will need to be composites of many different types of measures (44).

## Pain measurement in non-human animals

Despite developments in human pain research such as fMRI, animal models of pain have always been and continue to be necessary for ethical reasons and to obtain causal, mechanistic explanations of pain pathophysiology (45). As described in a prior, comprehensive review (46), algesiometry in laboratory animals has featured several waves of development. The classical assays featured the application of electrical, mechanical, or thermal stimuli to conveniently located body parts, producing pain for as long as it took for the stimulus to reach noxious intensity and the animal to reflexively or consciously withdraw. In the 1950s, several groups demonstrated that intraperitoneal injection of irritants (e.g., weak acids, phenylquinone) produced abdominal constriction (i.e., “writhing”) behavior (47–51) lasting 20–60 min, and that these assays appeared to have higher sensitivity to non-opioid analgesics like aspirin. In addition to lasting longer than the acute assays, these were tests of inescapable, suprathreshold pain (like clinical pain), and the endpoints (i.e., dependent measures) represented total time spent performing a behavior positively correlated with stimulus intensity rather than latency to a first response. Drawbacks included an uncertain location of the pain (visceral? muscle wall?), a non-linear stimulus-response relationship, and concerns over selectivity. An interesting advance occurred in 1977, when Dubuisson and Dennis (52), working in the laboratory of Ron Melzack (who in service of Stephen Dennis’ career declined to take an authorship), reported that formalin (i.e., diluted formaldehyde) injected into the forepaw of cats and rats produced guarding and licking/biting/shaking of the affected paw (In a classic footnote, they described the results of such an injection into their own finger.) In rats, a biphasic time course was noted, with early/acute (0–10 min) and late/tonic phases (>20 min) of pain behavior being interrupted by “a significant dip” lasting for about 10 min, and the two phases and “interphase” or “quiescent period” of the formalin test engendered voluminous study over the next few decades (see 53).

However, even the 60–90-min duration of the formalin test was clearly too short to properly model human chronic pain. A number of longer-lasting assays were developed, by injecting immune system-activating substances used previously to study inflammation—such as carrageenan (54), complete Freund's adjuvant (CFA) (55), zymosan (56), and urate crystals (57, 58)—into the hind paw or knee joint. A curious observation arising from the use of these assays was that the longer lasting the inflammation and the more time elapsed since injection, the less likely was the observation of any obvious nocifensive behaviors (i.e., licking, biting, shaking). For example, in the first paper reporting the effects of hind paw-injected CFA, Stein and colleagues (55) observed changes in body weight, food intake, core temperature, locomotion, defecation, and paw-pressure thresholds over 30 days post-injection, but no nocifensive behaviors. Thus, the advantage of a longer-lasting assay was paired with the disadvantage of needing to employ endpoints of questionable specificity to pain (e.g., hypolocomotion) or endpoints corresponding to comparatively minor human chronic pain symptoms, such as thermal or mechanical hypersensitivity (18, 38, 59).

A similar situation developed in the quest for longer-lasting animal models of neuropathic pain. The first such behavioral model was developed by Wall and colleagues (60), featuring a bizarre endpoint known as autotomy, whereby the animal progressively bites off the digits of the denervated paw. A credible model of phantom limb pain, this assay is almost never used because of its disagreeable aesthetics, controversial interpretation (61), and the fact that most human neuropathic pain is caused by partial, not complete injury to a nerve (62). In appreciation of this, Bennett and Xie (63) in 1988 developed an assay of peripheral mononeuropathy in which the sciatic nerve is slowly strangled by the placement of constrictive ligatures. Other strategies for producing partial disruptions of afferent input from the hind paw soon followed (64–66), and today there exist an alphabet soup of surgically and chemically induced nerve injuries (see 67). Although these differ in their symptom profile (e.g., only some featuring heat hypersensitivity), as with the inflammatory assays apart from (species-specific) guarding behavior there are no overt nocifensive behaviors to measure (68), and very little effect on activities of daily living (69, 70).

As measured by mechanical hypersensitivity, the most robust and thus most-used endpoint (71), the duration of pain varies in these assays. For example, hypersensitivity in the chronic constriction injury (CCI) assay is entirely resolved within 30–90 days post-surgery (63), whereas mechanical hypersensitivity in the spared nerve injury (SNI) lasts an entire lifetime (72). Bonica (73) defined chronic pain as that persists past normal healing time, but the need for a clearer cut off led Merskey (74) to propose (an arbitrary) 3 months as the definition of chronic, and this has been retained (75), although not without controversy (76). The SNI is suited for longer investigations, but 80% of extant preclinical pain studies are completed in less than 4 weeks post-injury (71). The wisdom of this *status quo* is called into question by two recent mouse studies showing important pathophysiological changes that do not occur until many months after injury (77, 78). It remains quite possible that 3 months in a mouse is…3 months.

Perhaps the strongest and most enduring criticism of the *status quo* in preclinical pain testing is the continued reliance on reflexive withdrawals to experimenter-delivered stimuli as an endpoint (e.g., 19, 79, 80). The most recent wave of development, therefore, has been to identify new endpoints. As described in recent, comprehensive reviews (81, 82), these can be broadly classified as pain-stimulated behaviors, pain-depressed behaviors, conditioned/motivated behaviors, measures of disability or quality of life, and biomarkers. Notable among these are: (1) operant conditioning to produce place avoidance (83); (2) conditioned place preference to analgesics as a way to infer pain during the classical conditioning (84, 85); (3) depression of previously favored activities such as wheel running (86), nest building (87), burrowing (88, 89), feeding (90), and cage-lid hanging (70); (4) measures of pain-related disability in, for example, grip strength (91); and (5) facial grimacing (92). Each of these have advantages and disadvantages compared to legacy endpoints, and many of these procedures are much more labor-intensive than their predecessors, although automation is proceeding apace (93). In some contexts, instead of behavioral endpoints one can employ non-behavioral proxies of nociceptive activity using powerful electrophysiological or calcium imaging techniques.

Finally, a recent development in preclinical algesiometry, especially at later stages of preclinical research, is to consider assessing new treatments *via* their effect on clinical pain states (e.g., arthritis, cancer pain) in companion animals (see 94, 95). Combined with more valid endpoints, such as automated measurement of grimacing in cats (96), this might represent a powerful way to predict clinically efficacy in human trials.

## The future of algesiometry

It can be argued that algesiometry in both humans and non-human animals have largely failed thus far in their respective aims. The continuing quest for objective biomarkers of pain in humans suggests that self-reported ratings are still not fully trusted, and many pain physicians increasingly avoid soliciting such ratings in favour of broader (but less selective) measures of functional disability and quality of life. Other dimensions of pain are being given more attention as well, including cognitive and social aspects (97), and there is likely to be much more study of “social pain”, aversive states not associated with physical injury and independent of somatic/visceral input (98). The repeated translational failures of analgesic development over the past decades have often, fairly or not, been blamed on the inadequacy of preclinical models (e.g., 99). Whether useful biomarkers and modern preclinical methods can improve the situation will be interesting to monitor over the next few decades. As ever, solutions to the measurement problem are critical for both the understanding and better management of pain.
